# Persistence of Toxic Activity of Fermentation Extracts from* Bacillus thuringiensis* var.* israelensis* after More Than Three Decades of Storage

**DOI:** 10.1155/2017/5402748

**Published:** 2017-11-20

**Authors:** Luis Jesús Galán-Wong, Rossana Gamiño-Hernández, David Fernández-Chapa, Graciela García-Díaz, Myriam A. De La Garza-Ramos, Claudio Guajardo-Barbosa, Hugo Alberto Luna-Olvera

**Affiliations:** ^1^Instituto de Biotecnología, Facultad de Ciencias Biológicas, Universidad Autónoma de Nuevo León, Ciudad Universitaria, 66450 San Nicolás de los Garza, NL, Mexico; ^2^Centro de Investigación/Facultad de Odontología, Universidad Autónoma de Nuevo León, Calle Dr. Eduardo Aguirre Pequeño y Silao s/n, Colonia Mitras Centro, 64460 Monterrey, NL, Mexico

## Abstract

This study was carried out to determine the persistence of toxicity of fermentation extracts of* Bacillus thuringiensis* var.* israelensis* after more than three decades of storage. For this purpose, a population of* Aedes aegypti* was established. The mortality rate of 20 spore-crystal extracts purified using the acetone-lactose coprecipitation method was measured and evaluated by bioassays according to a modified WHO protocol. The extracts with the highest mortality rate were determined in triplicate by their LD_50_ and LD_98_. All extracts showed toxicity at the highest tested dose (1000 ppm) and some, such as strains 3260 and 3501, still killed larvae at doses as low as 0.01 ppm. These data are surprising because no study on the activity of* B. thuringiensis* toxic proteins after such a long storage time has been reported.

## 1. Introduction

Mosquitoes today represent one of the most serious threats to public health because of their hematophagous nature and vector capacity. Some genera, such as* Aedes, Anopheles, *and* Culex,* transmit pathogens and parasites (viruses, protozoa, or nematodes) that cause devastating diseases such as dengue, Zika, chikungunya, encephalitis, yellow fever, malaria, and elephantiasis, which together are responsible for high morbidity and mortality in billions of people spread over almost half of the planet [1–6].

The need for more environmentally friendly insecticides and the increased resistance to chemicals have provided an excellent opportunity to eradicate mosquitoes with biological products based on* Bacillus thuringiensis* var.* israelensis (Bti)*. These bacteria conserve parasporal crystals and spores of the H-14 serotype, which when ingested by mosquito larvae are solubilized in the alkaline environment of the midgut. This leads to the activation of insecticidal proteins of the crystal which bind to receptors in the cell wall and form intestinal pores that result in larvae death. This insecticidal effect is produced by four toxins known as Cry4A, Cry4B, Cry11Aa, and Cyt1A. Their main attributes are high toxicity and specificity towards family Culicidae [7–9].

Since its discovery and description by Goldberg and Margalit [10],* Bti* has shown remarkable effectiveness in killing mosquitoes, in contrast to its innocuousness to humans and other mammals, aquatic vertebrates, invertebrates, and plants. Because of these characteristics, commercial preparations of these bacteria have been chosen for mosquito control programs [11–14]. Dulmage et al. [15] were among the most important pioneers in the development of technologies for the implementation of* Bti* as a biological pest control agent. They established diverse methodologies for mass production, product formulation, and power standardization. From these processes, several hundred fermentation extracts of cultures belonging to this microorganism, characterized by their insecticidal activity against Dipteran, were donated by the United States Department of Agriculture in 1989 to our research group. Since then, these extracts have been kept in the Universidad Autónoma de Nuevo Leon (UANL) International Collection of Entomopathogenic Bacilli. Particularly important within this collection are 20 fermentation extracts, which we decided to utilize in this research to prove their ability to kill mosquitoes under laboratory conditions through their residual activity.

## 2. Materials and Methods

### 2.1. *Bti *Strains

**Figure 1:**
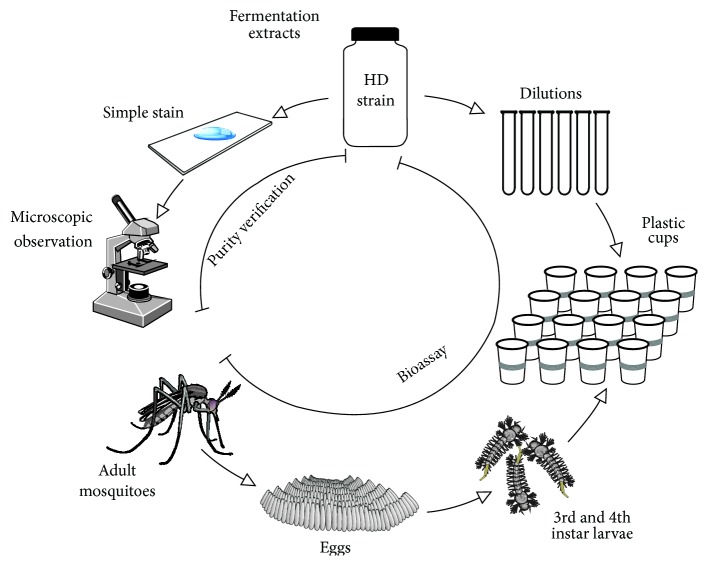
Diagram of experimental procedure.

*Bti* fermentation extracts from the collection of Dulmage et al. [15] recovered by lactose-acetone coprecipitation during the period from 1978 to 1983 were chosen. During this period, the extracts were stored in the dark in a dry space exclusively for this purpose at 25 ± 3°C in sterile and hermetic bottles. Twenty-four samples of the strain HD-500 and twenty-five of HD-567 were observed under the microscope with a simple Coomassie blue stain to ascertain the absence of contamination (Figure 1). They were then activated in Petri dishes and incubated in BD Bioxon nutrient agar for 72 hours to demonstrate their viability and, subsequently, samples that displayed viability were selected.

### 2.2. Mosquitoes

The larvae of the third and fourth instars of* A. aegypti* used in these experiments were obtained from the insectary of the Institute of Biotechnology of the School of Biology of the UANL. This mosquito colony is permanently maintained under pathogen-free conditions at 28 to 30°C with 60 to 80% relative humidity and light/dark cycles of 12 h. Under these conditions, the cycle from egg to adult is completed in three to four weeks. The larvae were fed daily with finely ground presterilized dog food (Pedigree brand). The pupae were removed daily to control the age of the mosquito and placed in a container that was introduced into a cage for 48 h for the emergence of adult mosquitoes. Cages of different sizes were used according to the magnitude of the mosquito population, which was fed daily with a 10% glucose solution soaked in cotton swabs and every 3 days with a blood source for 10 min. Oviposition of the females was stimulated with an ovitrap consisting of a water container covered with brown paper. Collected eggs were transferred to trays to perpetuate the cycle in such a way that larvae used in all the experiments were available.

### 2.3. Standard Preparation

As a positive control (100% mortality), a primary standard was prepared from VectoBac® 3000 UTI/mg (Valent BioSciences Corp. Libertyville, IL) at a concentration of 0.05 ppm. This was placed in Petri dishes with nutritive agar. Broth was taken and transferred to nutrient broth and incubated at 30°C at 150 rpm for 12 hours until an optical density greater than 1 (2 × 10^8^ CFU/ml), measured with a spectrophotometer at a wavelength of 540 nm, was reached. An aliquot of 1 ml in triplicate was added to 500 ml Erlenmeyer flasks with 100 ml of* Bti* medium (unpublished experimental medium) to verify the potency of the extracts. Distilled water was used as a negative control (Figure 1).

### 2.4. Bioassays

The World Health Organization guidelines for laboratory bioassays of bacterial larvicides were followed [16]. Suspensions of each fermentation extract were prepared at 2000 ppm in Erlenmeyer flasks. Dilutions were then made to obtain final concentrations of 1000, 100, 10, 1, 0.1, and 0.01, respectively. Transparent plastic cups with 250 ml capacity were used with 150 ml per vessel and 25 larvae of the second or third stage were added (Figure 1). To calculate LD_50_ of the fermentation extracts, three suspensions of 2%, 50%, and 98% were prepared. The positive control was performed in duplicate with the primary standard. Results were tabulated according to dose, considering the number of live and dead mosquitoes with 4 replications per treatment performed in triplicate at 24 hours. To calculate LD_50_, a linear regression was applied using the R statistical package, 2015 [17]. Bioassays were carried out at least three times and the validity of the results was evaluated with the primary extract obtained from VectoBac.

## 3. Results

**Figure 2:**
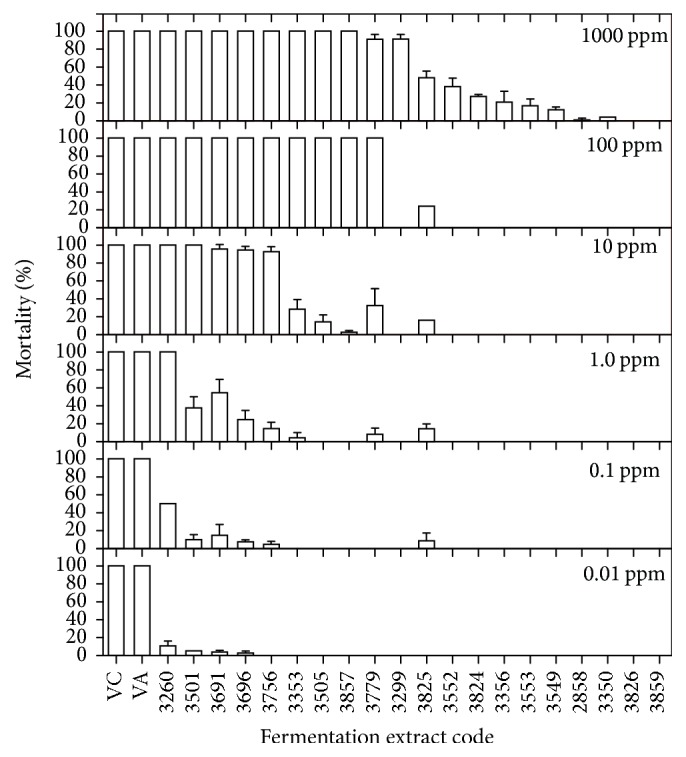
Toxicity of* Bacillus thuringiensis* var.* israelensis* fermentation extracts at different concentrations against* Aedes aegypti* (VC, commercial VectoBac; VA, activated VectoBac).

**Figure 3:**
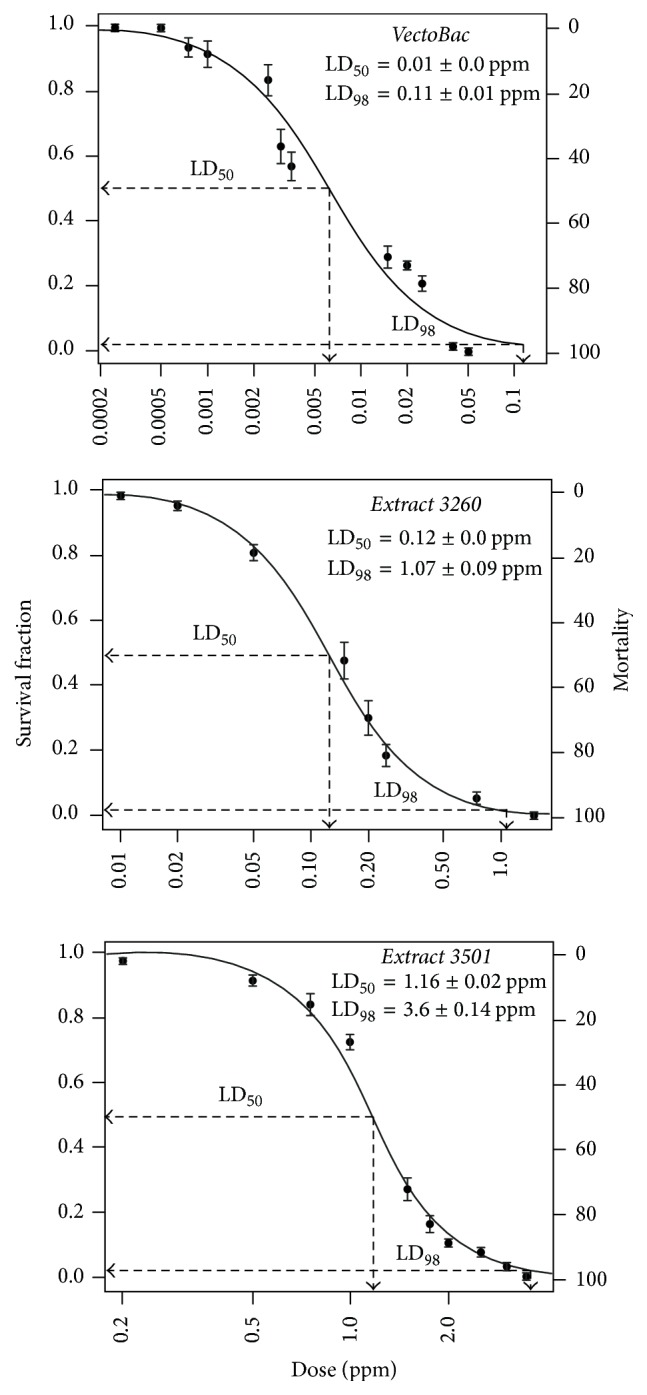
LD_50_ and LD_98_ of fermentation extracts 3501 and 3260 from* Bacillus thuringiensis* var.* israelensis*.

Of the 49* Bti* fermentation extracts from the collection, only 20 still contained spores as protein crystals; therefore, only these were used in the bioassays. Dead larvae were counted every 6 hours during a 24-hour period (Table 1). In this case, dying larvae were included to calculate the mortality rate. Most of these* Bti *extracts showed some toxic activity towards mosquito larvae in high concentrations, but their activity decreased as the dilution increased (Table 1). Thus, at 1000 ppm, up to 18 extracts managed to kill larvae to some degree; in fact, 10 extracts were 100% effective. This same trend was observed at 100 ppm, where only the extract from strain 3299 was unable to kill all test insects, but at 10 ppm only 5 of the extracts maintained this potency. An interesting fact was observed for the extract of strain 3260. Even at concentrations of 1 and 0.1, it maintained its toxic activity unaltered to kill all of the larvae. However, at a higher dilution (0.01 ppm), the extract was unable to maintain its residual activity (Figure 2). It is worth noting that the toxic activity of both the commercial product, VectoBac, and the laboratory preparation produced from it (VA) was maintained even at the lowest concentrations under our experimental conditions and together with strains 3260 and 3501 also showed a consistent ability to kill mosquito larvae at the lowest concentrations. Thus, LD_50_ and LD_98_ were 0.01 ± 0.0 and 0.11 ± 0.01; 0.12 ± 0.0 and 1.07 ± 0.09; and 1.16 ± 0.02 and 3.6 ± 0.14 ppm for VectoBac, 3260, and 3501, respectively (Figure 3).

## 4. Discussion

Our findings demonstrate the persistence of toxic activity of fermentation extracts of* Bti* against* A. aegypti *after three decades of storage. All extracts studied showed toxicity at the highest tested doses (1000 ppm) and some of the extracts, from strains 3260 and 3501, still killed larvae at doses as low as 0.01 ppm. Such data are surprising, since no study to date has reported the activity of* B. thuringiensis* toxic proteins after such a long storage time. On the other hand, the few reports on the persistence of toxicity in the field or under controlled conditions indicate that these proteins quickly lose their insecticidal properties due to different causes. Among the environmental factors that negatively affect the persistence of toxicity are temperature [18], solar radiation [19], pH [20], and organic matter [21]. However, other authors have shown that some conditions favor the possibility of increasing their persistence. For example, Saxena et al. [22] showed that Cry proteins from Bt maize root exudates maintained their larvicidal activity for more than 180 days in soil, while Tapp and Stotzky [23] reported this same toxic capacity more than 234 days after their incorporation into soil. This is probably a result of their binding to the clay particles of the porous matrix. In our case, the* Bti* protein crystals of the tested extracts were covered for more than three decades with the lactose matrix used during obtention of the fermentation extract. This suggests the important role that this disaccharide could have in maintaining the activity of the toxic proteins of the different* Bti* strains particularly stable after this period of time. In other varieties of this bacterium from the same collection of fermentation extracts (unpublished data), whose protective mechanism is still uncertain, the same effect does not appear. Although the activity of the extracts of* Bti* was found to be very low compared to treatment with VectoBac, it is notable that most maintained residual activity at the highest concentrations tested, and two of these extracts stood out at the minimum concentrations at which they could kill mosquito larvae. This is an encouraging sign regarding the possibility of improving the use of special strains and improved formulations to control insect vectors of diseases.

## 5. Conclusions

Fermentation extracts from* Bacillus thuringiensis* var.* israelensis* maintained their residual toxic activity against* Aedes aegypti* larvae after storage at 25 ± 3°C in the aforementioned conditions for more than three decades. Extracts 3260 and 3501 showed LD_50_ of 0.12 and 1.16 ppm, respectively. This work demonstrates that* Bti* protein crystals can prolong their toxicity in shelf life or field conditions under specific conditions.

## Figures and Tables

**Table 1:** Dead larvae counted every 6 hours after exposure to the extracts.

Treatment	Dead larvae count^*∗*^																													
	Concentration (ppm)/time (h)																													
	0.01					0.1					1					10					100					1000				
	1	6	12	18	24	1	6	12	18	24	1	6	12	18	24	1	6	12	18	24	1	6	12	18	24	1	6	12	18	24
Control^*∗∗*^	0	0	0	0	0	0	0	0	0	0	0	0	0	0	0	0	0	0	0	0	0	0	0	0	0	0	0	0	0	0
2858	0	0	0	0	0	0	0	0	0	0	0	0	0	0	0	0	0	0	0	0	0	0	0	0	0	0	0	0	0	0
3826	0	0	0	0	0	0	0	0	0	0	0	0	0	0	0	0	0	0	0	0	0	0	0	0	0	0	0	0	0	0
3859	0	0	0	0	0	0	0	0	0	0	0	0	0	0	0	0	0	0	0	0	0	0	0	0	0	0	0	0	0	0
3350	0	0	0	0	0	0	0	0	0	0	0	0	0	0	0	0	0	0	0	0	0	0	0	0	0	0	0	0	1	2
3549	0	0	0	0	0	0	0	0	0	0	0	0	0	0	0	0	0	0	0	0	0	0	0	0	0	0	0	2	3	4
3553	0	0	0	0	0	0	0	0	0	0	0	0	0	0	0	0	0	0	0	0	0	0	0	0	0	0	0	2	4	5
3356	0	0	0	0	0	0	0	0	0	0	0	0	0	0	0	0	0	0	0	0	0	0	0	0	0	0	0	2	4	6
3824	0	0	0	0	0	0	0	0	0	0	0	0	0	0	0	0	0	0	0	0	0	0	0	0	0	0	1	3	6	8
3552	0	0	0	0	0	0	0	0	0	0	0	0	0	0	0	0	0	0	0	0	0	0	0	0	0	0	1	2	6	10
3825	0	0	0	0	0	0	0	0	1	3	0	0	2	4	5	0	0	2	3	6	0	1	2	5	9	0	3	5	8	12
3299	0	0	0	0	0	0	0	0	0	0	0	0	0	0	0	0	0	0	0	0	0	0	0	0	0	2	8	14	19	23
3779	0	0	0	0	0	0	0	0	1	1	0	0	1	4	5	0	1	3	7	11	4	11	17	21	24	8	20	25	25	25
3353	0	0	0	0	0	0	0	0	0	0	0	0	1	1	2	0	2	6	9	10	0	6	15	22	25	11	23	25	25	25
3691	0	0	0	0	0	0	0	0	3	5	0	0	1	4	13	0	4	12	19	24	2	12	22	25	25	23	25	25	25	25
3505	0	0	0	0	0	0	0	0	0	0	0	0	0	0	0	0	0	2	4	5	8	20	25	25	25	25	25	25	25	25
3857	0	0	0	0	0	0	0	0	0	0	0	0	0	0	1	0	0	0	1	2	14	24	25	25	25	25	25	25	25	25
3756	0	0	0	0	0	0	0	0	1	1	0	0	2	4	5	4	9	18	21	24	13	25	25	25	25	25	25	25	25	25
3696	0	0	0	0	0	0	0	0	1	4	0	0	3	5	8	0	5	14	19	23	9	25	25	25	25	25	25	25	25	25
3501	0	0	0	0	0	0	0	0	2	6	0	2	6	10	13	14	24	25	25	25	25	25	25	25	25	25	25	25	25	25
3260	0	0	1	3	3	2	8	10	10	13	16	25	25	25	25	23	25	25	25	25	25	25	25	25	25	25	25	25	25	25
VA	16	25	25	25	25	25	25	25	25	25	25	25	25	25	25	25	25	25	25	25	25	25	25	25	25	25	25	25	25	25
VC	25	25	25	25	25	25	25	25	25	25	25	25	25	25	25	25	25	25	25	25	25	25	25	25	25	25	25	25	25	25

*∗* indicates average of four replications in three separate experiments; *∗∗* indicates distilled water. VA: activated VectoBac; VC: commercial VectoBac.
